# Maternal Exposure to Air Pollutants and Risk of Gestational Diabetes Mellitus in Taiwan

**DOI:** 10.3390/ijerph14121604

**Published:** 2017-12-20

**Authors:** Hsiu-Nien Shen, Sheng-Yuan Hua, Chang-Ta Chiu, Chung-Yi Li

**Affiliations:** 1 Department of Intensive Care Medicine, Chi Mei Medical Center, Yong-Kang District, Tainan City, Taiwan; hsiunian@gmail.com; 2 Department of Public Health, College of Medicine, National Cheng Kung University, Tainan City, Taiwan; azureluno@hotmail.com; 3 Department of Dentistry, An Nan Hospital, China Medical University, Tainan City, Taiwan; 4 Department of Public Health, College of Public Health, China Medical University, Taichung City, Taiwan

**Keywords:** air pollution, gestational diabetes mellitus, nested case-control study, dose-response relationship

## Abstract

Mounting evidence has shown an increased risk of gestational diabetes mellitus (GDM) in association with elevated exposure to air pollution. However, limited evidence is available concerning the effect of specific air pollutant(s) on GDM incidence. We conducted this case-control study on 6717 mothers with GDM diagnosed in 2006–2013 and 6717 age- and year of delivery-matched controls to further address the risk of GDM in relation to specific air pollutant. Both cases and controls were selected from a cohort of 1-million beneficiaries of Taiwan’s National Health Insurance program registered in 2005. Maternal exposures to mean daily air pollutant concentration, derived from 76 fixed air quality monitoring stations within the 12-week period prior to pregnancy and during the 1st and 2nd trimesters, were assessed by the spatial analyst method (i.e., ordinary kriging) with the ArcGIS software. After controlling for potential confounders and other air pollutants, an increase in pre-pregnancy exposure of 1 inter-quartile range (IQR) for PM_2.5_ and SO_2_ was found to associate with a significantly elevated odds ratio (OR) of GDM at 1.10 (95% confidence interval (CI) 1.03–1.18 and 1.37 (95% CI 1.30–1.45), respectively. Exposures to PM_2.5_ and SO_2_ during the 1st and 2nd trimesters were also associated with significantly increased ORs, which were 1.09 (95% CI 1.02–1.17) and 1.07 (95% CI 1.01–1.14) for PM_2.5_, and 1.37 (95% CI 1.30–1.45) and 1.38 (95% CI 1.31–1.46) for SO_2_. It was concluded that higher pre- and post-pregnancy exposures to PM_2.5_ and SO_2_ for mothers were associated with a significantly but modestly elevated risk of GDM.

## 1. Introduction

Although the mechanism by which air pollution mediates propensity to diabetes onset is not fully understood, growing evidence accumulated over the past decade tends to suggest a link between higher air pollution exposure and elevated risk of diabetes. However, most of the studies focused on the influence of air pollution on type 2 diabetes mellitus (T2DM). Balti et al. [1] conducted a meta-analysis of five prospective studies and found that the overall effect on T2DM incidence was significant for both nitrogen dioxide (NO_2_) and particulate matter ≤2.5 μm in diameter (PM_2.5_), with an increased risk of 13% and 11%, respectively. Later updated meta-analyses further reported that per 10 μg/m^3^ increase in NO_2_ exposure was significantly associated with an 8% increase in T2DM risk [2]; and the increased risk of future T2DM associated with exposure to 10 μg/m^3^ increase of PM_2.5_ was estimated in a range of 10% to 27% [2,3].

Compared to the results of studies on the air pollution and T2DM relationship, findings from studies of the association of air pollution with risk of gestational diabetes mellitus (GDM) have been neither comprehensive nor consistent. Hooven et al. [4] conducted the first epidemiological study in The Netherlands to address the relationship between air pollution and GDM, which found no association between several proxies of air pollution (e.g., traffic intensity and distance to major roads) and GDM incidence. Similar results were observed in a Japanese study by Yorifuji et al. [5]. On the other hand, A Swedish study by Malmqvist et al. [6] found that higher exposure to NO_x_ (at the 3rd and 4th quartiles) during the 1st trimester was associated with a significantly elevated risk of GDM, with an odds ratio (OR) of 1.52 and 1.69, respectively. Additionally, several studies conducted in the USA have consistently reported positive associations of GDM or impaired glucose tolerance (IGT) with various air pollutants including PM_2.5_ [7,8,9], NO_x_ [6,10], SO_2_ [10], and O_3_ [8]. The magnitude of relative risk for GDM or IGT in relation to air pollution noted in these US studies varied greatly from 1.05 (per 1 inter-quartile-range (IQR) increase in pre-conception exposure to SO_2_ and risk of IGT) [10] to 2.63 (exposure to PM_2.5_ at the highest quartile during the 2nd trimester and risk of GDM) [9]. A recent Taiwanese study noted that the risk of GDM was significantly but only slightly (OR, 1.05) increased in women who had NO exposure during the first and second trimesters [11].

Limitations of the current epidemiological findings and evidence regarding the association of air pollution and risk of GDM include failure to simultaneous adjust for the other air pollutants in the analysis, utilization of different time periods for air pollution exposure assessment, and incomplete adjustment for known risk factors for GDM such as maternal socioeconomic background. Moreover, limited data are available for non-Western populations, given previous studies showed apparent ethnic variation in GDM incidence [12].

Outdoor air pollution is a major environmental health problem affecting everyone in developed and developing countries. Additionally, GDM has been related to substantial short- and long-term adverse health outcomes, such as increased risk of developing cardio-metabolic disorders later in life among both women and their offspring [13]. Moreover, the global burden of GDM could be overlooked given a high GDM prevalence globally, ranging from 5.8% (1.8–22.3%) in Europe to 12.9% (8.4–24.5%) [14]. Our study aimed to investigate the associations of GDM incidence in association with pre- and post-pregnancy exposure to various air pollutants taken into account simultaneously.

## 2. Materials and Methods

### 2.1. Research Data

Data analyzed in this study were retrieved from Taiwan’s National Health Insurance Research Data (NHIRD) and the air pollutant concentration data were obtained from the monitoring data supervised by Environmental Protection Administration of Taiwan. Our access to the NHIRD was approved by the Review Committee of the National Health Research Institutes. The study was also approved by the Research Ethics Committee of the National Cheng Kung University (approval number 103-010). The NHIRD were retrieved from Taiwan’s National Health Insurance (NHI) program, which enrolls >99% of Taiwanese residents [15]. The NHIRD cover all medical claims from nearly all hospitals and clinics in Taiwan. Each claim data are involved with patients’ demographic characteristics, disease diagnostic codes, prescription records, and medical expenditures. In the present study, we used inpatient and outpatient medical claims of a representative sample of one million beneficiaries randomly selected from all beneficiaries registered in 2005.

### 2.2. Study Design and Participants

We conducted a case-control study nested within the one million people mentioned above. Between 2006 and 2013, a total of 63,177 singleton deliveries given by 36,434 mothers were noted. Among them, 11,688 singleton deliveries had a GDM diagnosis (International Classification of Diseases, Ninth Revision, Clinical Modification, ICD-9-CM code: 648.0 or 648.8) in mothers at discharge. We aggregated these 11,688 singleton deliveries into 7240 mothers, and retained the claim data of the first-time delivery for each mother who had more than 1 delivery in 2006–2013. We further excluded 523 mothers who had a history of diabetes (ICD-9-CM code: 250.xx) or GDM between 1 January 1997 and date of the first-time singleton delivery in 2006–2013, leaving 6717 mothers considered as the cases of newly diagnosed GDM. One control mother was randomly selected for each case, by matching case mother on year and age at delivery. The controls should be free from inpatient or outpatient diagnosis of diabetes or GDM between 1 January 1997 and 31 December 2013. Totally, 6717 control mothers were selected.

### 2.3. Assessment of Exposure to Air Pollution

Air pollution data were collected from all 76 fixed-site air quality monitoring stations (AQMSs) supervised by the Taiwan Environmental Protection Agency during 2005–2013. We managed to exclude those 10 AQMSs located in industrial parks or remote areas where very few people lived, but found no apparent difference in air pollutant concentration estimated. Thus, we included air pollution data of all AQMSs in this analysis. At each AQMS, the concentration was recorded hourly for each of the following air pollutants: particulate matters (PM) with a diameter of 10 μm or less (PM_10_, μg/m^3^), PM with a diameter of 2.5 μm or less (PM_2.5_, μg/m^3^), sulfate dioxide (SO_2_, ppb), ozone (O_3_, ppb), nitrogen dioxide (NO_2_, ppb), and carbon monoxide (CO, ppm) [16].

The hourly data recorded at each AQMS between 2005 and 2013 were further averaged into daily mean concentration for each air pollutant. We retrospectively assessed maternal daily mean exposure to various air pollutants during the 12-week period prior to pregnancy, the first trimester (1st–12th week), and the second trimester (13th–24th week) respectively. In Taiwan, the NHI provides free prenatal care and recommends ten prenatal visits for all pregnant women in order to reduce the risk of poor pregnancy outcomes and to decrease the need for pediatric care after birth [17]. With the information of gestational age and delivery date of each infant available in the NHIRD, we were able to estimate the date of conception for each pregnant woman.

Air pollutant concentration was estimated for the center point coordinator of each of the 316 cities/townships all over Taiwan by the spatial analyst method (i.e., ordinary kriging) with the ArcGIS Desktop v.10 software (ESRI Inc., Redlands, CA, USA), which was frequently used in previous studies [18,19,20]. This spatial interpolation and cross-validation approach interpolates exposure concentration to a regular grid (250 × 250 m) across Taiwan. The cross-validation was based on the pollutant data of those stations within 3 km outside of the city/township boundary. Because the NHIRD include no information of study subjects’ moving during the gestation, we used only the residential city/township on date of delivery for air pollution exposure assessment.

### 2.4. Potential Confounders

Apart from matching variables (e.g., age and year at delivery), we considered some other maternal characteristics and co-morbidity presumably associated with risk of GDM in the analysis, including season of delivery [21], number of births [22], obesity [23], history of polycystic ovary syndrome (PCOS) [24,25], and disease burden indicated by Charlson’s Co-morbidity Index (CCI) [26]. In addition, we also considered personal monthly income and city/township specific median family income in the analysis, as previous studies reported that lower socioeconomic status may increase risk of GDM [27,28]. We also adjusted for city/township level of urbanization to minimize the potential confounding by differential accessibility and availability of medical care, as well as to account for the possible urban–rural difference in quality of diagnostic techniques [29].

### 2.5. Statistical Analysis

We first compared the characteristics between cases and controls. Descriptive statistics of air pollutants’ concentration were calculated between cases and controls, according to pre- and post-pregnancy periods. Additionally, Pearson’s correlation coefficients were calculated to indicate the strength of pair-wise associations of concentration among air pollutants. The Pearson’s correlation coefficients for the association between PM_2.5_ and PM_10_ (*r* = 0.963–0.964), and the association between NO_2_ and CO (*r* = 0.934–0.937) were so strong during the pre- and post-pregnancy periods. Thus, we assessed the risk of GDM in associations with only four air pollutants (namely, PM_2.5_, SO_2_, O_3_, and NO_2_) in order to avoid the potential problem of co-linearity. We calculated, using conditional logistic regression model, crude and covariate adjusted ORs to estimate the relative risk of GDM in relation to specific air pollutant determined at various pre- and post-pregnancy periods. The potential confounders adjusted in the multivariate regression model included all variables listed in Table 1 and all other air pollutants.

All statistical analyses were performed with SAS (version 9.3; SAS Institute, Cary, NC, USA). A *p*-value < 0.05 was considered statistically significant.

## 3. Results

While more cases than controls were not primiparae (47.0% vs. 32.5%), cases and controls were essentially at the same age of delivery. The season of delivery was not similarly distributed between cases and controls, in which more cases gave births during the fall and winter. Cases were less likely than controls to be actively employed; and were more likely to have lower monthly income, live in rural areas, and reside in city/townships with lower median family income. Additionally, the prevalence of diagnosed PCOS (7.5% vs. 5.2%) and obesity (1.2% vs. 0.7%) was significantly higher in cases than in controls. Moreover, cases also had a significantly greater CCI score than controls (0.47 vs. 0.41) (Table 1).

**Figure 1:**
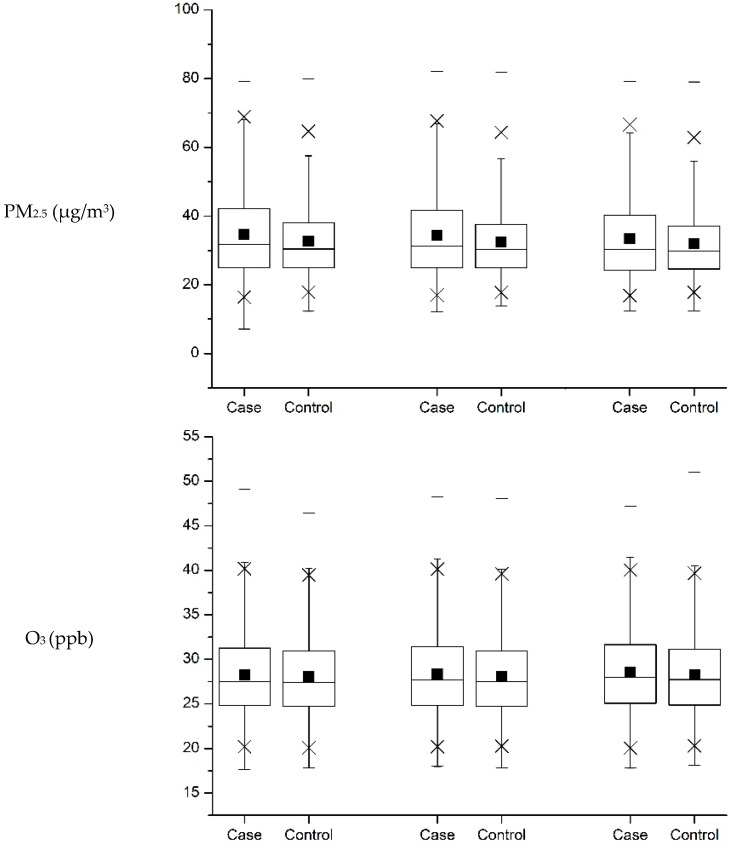

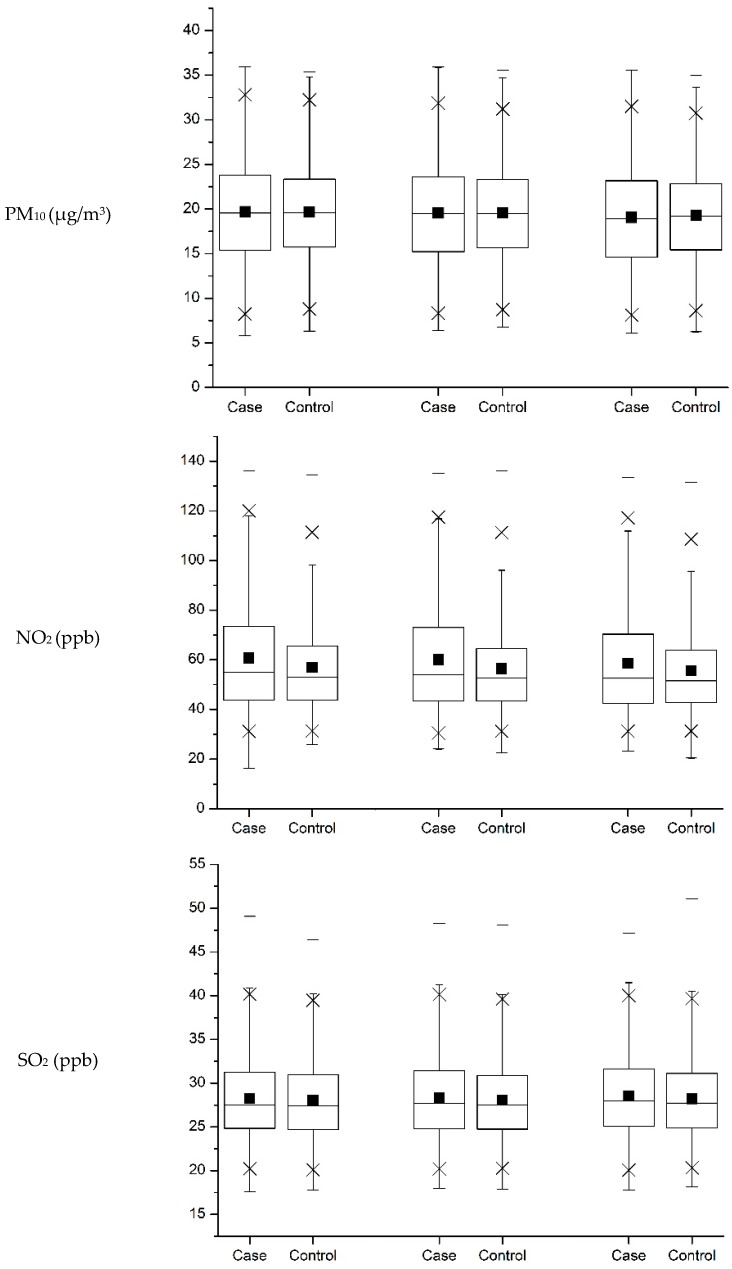

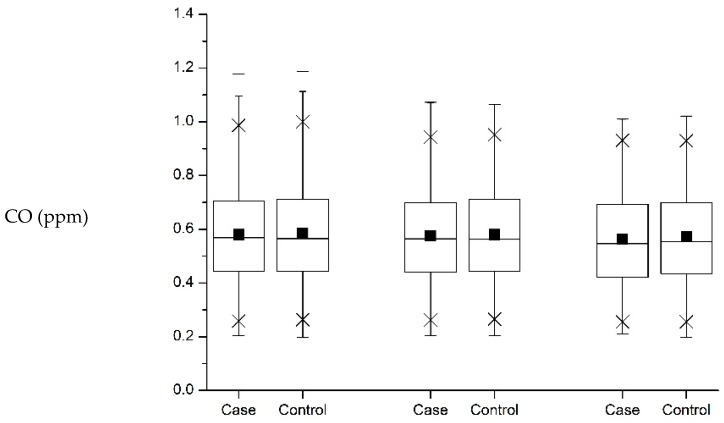
Pre- and post-pregnancy average daily exposure to various air pollutants for cases and controls. Note: For the graph of reach pollutant. -: Min. and Max.; ×: 1% and 99%; ◼: Mean. The 12-week period before pregnancy (**left**); The 1st trimester (**middle**); The 2nd trimester (**right**).

Supplemental Table 2 shows the mean concentration of various air pollutants determined at various periods before and after pregnancy for cases and controls. Cases had apparently higher pre- and post-pregnancy exposures to PM_2.5_, PM_10_, and SO_2_. On the other hand, the mean daily exposures to O_3_, NO_2_, and CO were essentially similar between cases and controls over time. Figure 1 compares the boxplots of concentration between cases and control for various air pollutants over the pre- and post-pregnancy periods.

Table 2 shows crude and adjusted OR of GDM in association with pre- and post-pregnancy exposures to various air pollutants.

We noted that per 1 IQR increase in pre-pregnancy exposures to PM_2.5_ and SO_2_ was associated with a significantly increased risk of GDM, with a covariate adjusted OR of 1.10 (95% confidence interval (CI) 1.03–1.18) and 1.37 (95% CI 1.30–1.45), respectively. The significantly elevated adjusted OR associated with a 1 standard deviation (SD) increase in pre-pregnancy exposures to PM_2.5_ and SO_2_ was 1.08 (95% CI: 1.03–1.14) and 1.32 (95% CI: 1.26–1.38), respectively. Pre-pregnancy exposures to O_3_ and NO_2_ were not significantly associated with risk of GDM. Similar results were observed for air pollution exposures during the first and second trimesters.

## 4. Discussion

With this large-scale study that included a representative sample of Taiwanese mothers, we found that exposures to PM_2.5_ and SO_2_ during the pre-conception and early pregnancy periods were associated with a modest but significantly increased risk of GDM. On the other hand, both O_3_ and NO_2_ posed no significant influence on risk of GDM.

PM_2.5_ is one of the air pollutants most frequently investigated and noted to pose influences on risks of IGT or GDM [7,8,9]. Unlike NO_2_ and SO_2_ which are mostly emitted from motor vehicles and combustion of coal in manufacturing factories, respectively, PM_2.5_, on the other hand, has been released from multiple sources, including fossil-fuel combustion by motor vehicles and stationary sources such as power plants. Apart from the petrochemical airborne effluents, a recent Taiwanese study estimated that coal combustion, iron ore and steel industry, and non-ferrous metallurgy accounted for some 70% of the primary PM_2.5_ in Taiwan [30]; and that these particles have the capacity to deposit in the lungs. Compared to the first quartile exposure to PM_2.5_ (8.5–10.8 μg/m^3^), Fleisch et al. [7] found women with the highest quartile exposure (12.8–15.9 μg/m^3^) during the 2nd trimester had a 2.63 (95% CI 1.15–6.01) times higher risk of having IGT. Additionally, based on more than 14,000 women with GDM, Hu et al. [8] found that per 5 μg/m^3^ increase in PM_2.5_ exposure during the first and second trimester was associated with a significantly elevated risk of GDM, with an OR of 1.16 (95% CI 1.11–1.21) and 1.15 (95% CI 1.10–1.22), respectively. Although Fleisch et al. [9] found that none of the residential exposures in a range of 1.3–19.3 μg/m^3^ over the second trimester were associated with GDM (OR = 0.99, 95% CI 0.95, 1.03) for each IQR increment, they did note that women less than 20 years had 1.36 higher odds of GDM (95% CI 1.08, 1.70) for each IQR increment in PM_2.5_ exposure at the 2nd trimester. Our study was essentially consistent with the above previous studies, but with a somewhat smaller magnitude (≤10%) in relative risk estimates. Our study additionally took into account certain risk factors for GDM including obesity, PCOS, and socioeconomic status of mothers, which were not considered in previous studies.

SO_2_ was the other air pollutant that showed significant adverse effects on GDM across the pre- and post-pregnancy periods in our study. The increase in risk of GDM in relation to per IQR or 1 SD increment in exposure was greater for SO_2_ (31–38%) than for PM_2.5_ (<10%). Robledo et al. [10] also found significant associations of GDM with IQR increment in the pre-conception (5.37 ppb) and 1st trimester (3.31 ppb) periods, with an OR of 1.05 (95% CI 1.01–1.09) and 1.04 (95% CI 1.01–1.08). Unlike some previous studies that noted increased risks of GDM in relation to NOx [6,10] and NO [11] exposures, our study did not find a significant association between NO_2_ exposure and risk of GDM.

Although the mechanisms that potentially link air pollution to GDM have not been fully elucidated, there are a number of possible biological pathways linking air pollutants to diabetes, mainly T2DM. These pathways could include endothelial dysfunction, dysregulation of the visceral adipose tissue through inflammation, hepatic insulin resistance, elevated hemoglobin A1c (HbA1c) level, elevated blood pressure, and alterations in autonomic tone, which may increase insulin resistance [3]. Both experimental and epidemiologic studies suggest that environmental exposures to air pollutants can increase the risk of insulin resistance, which may in turn lead to an obvious link between air pollution and GDM [31]. A recent study found positive associations between PM_2.5_ and oral glucose tolerance test (OGTT) glucose levels, an indicator of insulin resistance, during pregnancy. The association was especially pronounced for the fasting and 1-h glucose levels [32]. The influence of glucose homeostasis during pregnancy posed by PM_2.5_ could further increase the risk of glucose imbalance since pregnancy itself is a complex metabolic adaptation process including impaired glucose homeostasis, which may increase a woman’s susceptibility to air pollution [32,33]. Inflammation is another major potential mechanism that could explain the pathogenesis underlying the association between air pollution and GDM. There is high possibility for the activation of the inflammatory pathway and oxidative stress pathway by air pollutants in particular fine particles [31,34].

The present study broadens our understanding that certain ambient air pollutants (PM_2.5_ and SO_2_) may increase risk of GDM in women, which is of important clinical and preventive implications in Taiwan where the PM_2.5_ concentration is much higher than many parts of the world. For example, Fleisch et al. [9] reported residential exposures in a range of 1.3–19.3 μg/m^3^ in Massachusetts. In addition, a recent study showed an annual mean PM_2.5_ of 11.3 and 13.6 μg/m^3^ in Denmark and Austria, respectively [35]. Our data showed a mean PM_2.5_ of some 34 and 32 μg/m^3^ for cases and controls, respectively. Although the main sources of outdoor air pollution are well beyond the control of individuals, pregnant women should still be informed of minimizing as much exposure as possible to air pollution. Besides, the public healthcare systems in Taiwan should aim to mitigate the occurrence of GDM through healthcare system preparedness, screening for impaired glucose and timely warnings, medical advice, and health education.

This study has some limitations. First, the locations of AQMSs may not necessarily reflect air pollution levels in inhabited areas despite the utilization of modelling techniques, which might have biased our study results and attenuated the true relationship between air pollution and GDM. The potential for exposure information bias could further be introduced by our incapability of obtaining the detailed information of study subjects’ mobility during the pre- and post-pregnancy periods. The potential exposure misclassification is likely to be non-differential because the likelihood of living close to AQMSs or moving during the pregnancy should be independent of having a diagnosis of GDM. Second, although we managed to exclude the influence of obesity and PCOS known to predispose DKA incidence, the residual influence by some other risk factors for GDM might exist, as Taiwan’s NHI claim data include no information on blood work data, anthropometric parameters (e.g., body mass index) and diet. Greater body mass index, higher cholesterol [36,37] and deficiency in vitamin D [38] have been considered to increase risk of GDM. Additionally, we also had no information on family history of diabetes, which is also a major risk factor for GDM [39]. Despite a lack of information on the above risk factors for GDM, the potential for these uncontrolled variables to incur confounding could be small as we considered personal monthly income and residential city/township-specific family income in the analyses. These uncontrolled variables including BMI, diet, lifestyle, and diabetes are greatly related to socioeconomic status. Third, we aimed to examine the specific period before and after pregnancy within which air pollution is most etiologically relevant, if any, to the incidence of GDM. Because the inter-correlations of air pollution levels in the three pre- and post-pregnancy study periods were high, the relative risk of GDM in relation to air pollution for the three time periods were very similar. In analyzing the risk of GDM for a specific time period, we managed but failed to control for air pollution levels in the other two study periods mainly due to the problem of co-linearity among air pollution levels across the three time periods. This statistical problem has limited specific interpretations of our findings regarding which time period within which the air pollution exposure is most relevant to the risk of GDM.

## 5. Conclusions

This study noted a modest but statistically increased risk of GDM in women with higher exposures to PM_2.5_ and SO_2_ during the 12-week pre-conception period, as well as during the first two trimesters of pregnancy. Additionally, like many other nations, Taiwan’s Environmental Protection Agency also uses the Pollutant Standards Index, or PSI, to indicate the air quality. Because the PSI is based on six pollutants, including PM_10_, PM_2.5_, SO_2_, CO, O_3_, and NO_2_, it may not necessarily reflect the air pollution level of specific air pollutant. Therefore, pregnant women and clinicians are advised not to use PSI as the only reference to take measures for reducing air exposure. Instead, the pollution levels of particulate matters and sulphur dioxide may be more relevant to the risk of GDM.

## Figures and Tables

**Table 1:** Characteristics of cases and controls.

	Cases		Controls		*p*-Value ^a^
	*n*	*%*	*n*	*%*	
Age at delivery, years					
<25	468	7.0	470	7.0	
25–29	1782	26.5	1780	26.5	
30–34	2946	43.9	2949	43.9	
>35	1521	22.6	1518	22.6	
Mean ± SD	31.30 ± 4.54		31.12 ± 4.51		0.0042
Primipara					<0.0001
Yes	3560	53.0	4532	67.5	
No	3157	47.0	2185	32.5	
Monthly income, NTD					<0.0001
Dependent	1484	22.1	1355	20.2	
≤15,840	981	14.6	959	14.3	
15,841–22,800	1854	27.6	1833	27.3	
22,801–28,800	719	10.7	749	11.2	
28,801–36,300	725	10.8	733	10.9	
36,301–45,800	672	10.0	718	10.7	
>45,800	282	4.2	370	5.5	
Urbanization					<0.0001
Urban	1847	27.5	1855	27.6	
Satellite	2613	38.9	3081	45.9	
Rural	2257	33.6	1781	26.5	
Township-specific median family annual income quartiles ^a^, NTD					<0.0001
≤Q1	1686	25.1	1374	20.5	
>Q1–Q2	1679	25.0	1647	24.5	
>Q2–Q3	1679	25.0	1753	26.1	
>Q3	1673	24.9	1943	28.9	
Mean ± SD	873,000 ± 224,000		893,000 ± 228,000		0.0143
Diagnosed co-morbidity					
Polycystic ovary syndrome	504	7.5	3509	5.2	<0.0001
Obesity	82	1.2	47	0.7	<0.0001
Charlson Comorbidity Index					
0	4442	66.1	4628	68.9	<0.0001
1	1680	25.0	1605	23.9	
2	442	6.6	363	5.4	
≧3	153	2.3	121	1.8	
Mean ± SD	0.47 ± 0.80		0.41 ± 0.74		
Season of delivery					0.0143
Spring (March–May)	1545	23.0	1619	24.1	
Summer (June–August)	1587	23.6	1652	24.6	
Fall (September–November)	1888	28.1	1780	26.5	
Winter (December–February)	1697	25.3	1666	24.8	
Total	6717		6717		

SD, standard deviation; NTD New Taiwan Dollars, 1 USD ≅ 30 NTD; ^a^ Q1 = 730,000, Q2 = 803,000, Q3 = 944,000.

**Table 2:** Crude and adjusted odds ratio of gestational diabetes mellitus in association with various air pollutants exposure before and after pregnancy.

Time Period and Increase in Exposure	Odds Ratio (95% CI)	
	Crude	Adjusted ^a^
*Within the 12-week period prior to pregnancy*		
PM_2.5_ (μg/m^3^)		
Per 1 IQR increase	1.22 (1.17–1.26)	**1.10 (1.03–1.18) ^b^**
Per 1 SD increase	1.17 (1.13–1.21)	**1.08 (1.03–1.14)**
SO_2_ (ppb)		
Per 1 IQR increase	1.28 (1.24–1.33)	**1.37 (1.30–1.45)**
Per 1 SD increase	1.24 (1.21–1.28)	**1.32 (1.26–1.38)**
O_3_ (ppb)		
Per 1 IQR increase	1.08 (1.03–1.13)	1.01 (0.96–1.07)
Per 1 SD increase	1.05 (1.02–1.09)	1.01 (0.97–1.05)
NO_2_ (ppb)		
Per 1 IQR increase	1.01 (0.96–1.06)	0.96 (0.90–1.02)
Per 1 SD increase	1.01 (0.97–1.04)	0.97 (0.93–1.02)
*During the 1st trimester*		
PM_2.5_ (μg/m^3^)		
Per 1 IQR increase	1.20 (1.16–1.25)	**1.09 (1.02–1.17)**
Per 1 SD increase	1.16 (1.13–1.20)	**1.08 (1.02–1.13)**
SO_2_ (ppb)		
Per 1 IQR increase	1.28 (1.23–1.32)	**1.37 (1.30–1.45)**
Per 1 SD increase	1.23 (1.19–1.27)	**1.31 (1.25–1.37)**
O_3_ (ppb)		
Per 1 IQR increase	1.07 (1.03–1.13)	1.02 (0.96–1.08)
Per 1 SD increase	1.05 (1.02–1.09)	1.01 (0.97–1.06)
NO_2_ (ppb)		
Per 1 IQR increase	1.00 (0.95–1.05)	0.93 (0.88–1.00)
Per 1 SD increase	1.00 (0.97–1.03)	0.95 (0.91–1.00)
*During the 2nd trimester*		
PM_2.5_ (μg/m^3^)		
Per 1 IQR increase	1.16 (1.12–1.21)	**1.07 (1.01–1.14)**
Per 1 SD increase	1.13 (1.10–1.17)	**1.06 (1.01–1.11)**
SO_2_ (ppb)		
Per 1 IQR increase	1.27 (1.22–1.31)	**1.38 (1.31–1.46)**
Per 1 SD increase	1.23 (1.19–1.26)	**1.32 (1.26–1.38)**
O_3_ (ppb)		
Per 1 IQR increase	1.11 (1.06–1.16)	1.04 (0.99–1.11)
Per 1 SD increase	1.07 (1.04–1.11)	1.03 (0.99–1.07)
NO_2_ (ppb)		
Per 1 IQR increase	0.95 (0.91–0.99)	0.97 (0.93–1.02)
Per 1 SD increase	0.97 (0.94–0.99)	0.98 (0.93–1.03)

^a^ Covariates adjusted are variables listed in Table 1; ^b^ The bold numbers of adjusted ORs are indicative of statistical significance at an α-level of 0.05.
